# The pupylation pathway and its role in mycobacteria

**DOI:** 10.1186/1741-7007-10-95

**Published:** 2012-11-30

**Authors:** Jonas Barandun, Cyrille L Delley, Eilika Weber-Ban

**Affiliations:** 1 ETH Zurich, Institute of Molecular Biology & Biophysics, CH-8093 Zurich, Switzerland

## Abstract

Pupylation is a post-translational protein modification occurring in actinobacteria through which the small, intrinsically disordered protein Pup (prokaryotic ubiquitin-like protein) is conjugated to lysine residues of proteins, marking them for proteasomal degradation. Although functionally related to ubiquitination, pupylation is carried out by different enzymes that are evolutionarily linked to bacterial carboxylate-amine ligases. Here, we compare the mechanism of Pup-conjugation to target proteins with ubiquitination, describe the evolutionary emergence of pupylation and discuss the importance of this pathway for survival of *Mycobacterium tuberculosis *in the host.

## 

Post-translational protein modification is a prevalent means of diversification and regulation in all cells [1]. The functional consequences range from immediate effects like changes in protein conformation or stability, regulation of enzymatic activities to the determination of subcellular localization. Tags marking substrates for degradation by energy-dependent protease complexes exist in pro- and eukaryotes, as exemplified by eukaryotic ubiquitination [2,3] or bacterial co-translational ssrA-tagging [4]. However, until recently, the use of small-protein modifiers such as ubiquitin was considered a feature exclusive to eukaryotic cells. The discovery of pupylation, the covalent modification of protein lysines with prokaryotic, ubiquitin-like protein Pup, in *Mycobacterium tuberculosis *(*Mtb*) and *Mycobacterium smegmatis *[5,6] and the detection of conjugates between small archaeal modifier proteins (SAMPs) and substrate lysines in archaea [7,8] show that prokaryotes also employ macromolecular tags. It has been demonstrated that modification of target proteins with Pup occurs by a chemical pathway distinct from ubiquitination [9] (Figure 1). However, like ubiquitination, tagging with Pup can render proteins as substrates for proteasomal degradation [5,6,10]. The existence of a depupylation activity in actinobacteria [11,12] and the fact that some members harbor the pupylation gene locus without encoding proteasomal subunits suggest that pupylation might fulfill a broader role in regulation and cellular signaling. The purpose of the pupylation system in actinobacteria is still a matter of investigation. In *Mtb*, the Pup-proteasome system (PPS) has been linked to the bacterium's survival strategy inside the host macrophages [13,14].

**Figure 1:**
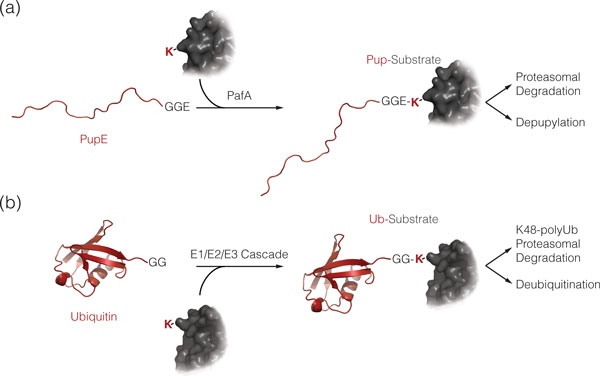
**Bacterial pupylation, like eukaryotic ubiquitination, targets proteins for proteasomal degradation**. **(a,b) **Pupylation (a) or ubiquitination (b) of a target protein is shown. Both small protein modifiers (red) are attached to a lysine side chain of a substrate protein (grey). A random coil model of Pup (red) represents its intrinsically disordered state in solution. In contrast, ubiquitin (Ub) adopts a stable β-grasp fold (PDB 1aar). Note that Ub is linked to the substrate lysine via its carboxy-terminal di-glycine-motif ('GG'), while Pup is attached via its carboxy-terminal glutamate ('GGE').

## An ubiquitin-like modification pathway in bacteria marks proteins for proteasomal degradation

**Figure 2:**
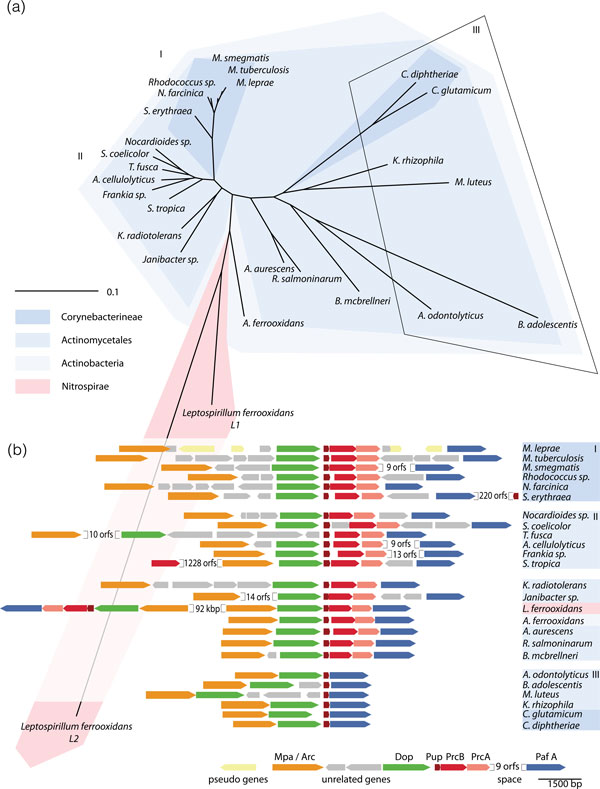
**Occurrence, genomic organization and relatedness of the pupylation gene locus**. **(a) **Phylogenetic analysis of the combined Arc, Dop, Pup and PafA amino acid sequences reveals tight clustering of proteasome-harboring members (clusters I and II), whereas members without proteasomal genes in the pupylation locus exhibit much greater sequence variation (cluster III). The pupylation enzymes of *Leptospirillum ferrooxidans*, a Nitrospirae exponent, likely originate in a member of the acidimicrobiales, a subclass of the actinobacteria. **(b) **Genomic context of the pupylation-relevant enzymes. The genomes are listed counter clockwise as they appear in (a). The enzymatic order in the genome is rigidly conserved through all pupylation-competent organisms, although some species exist (as *Saccharopolyspora erythraea *or *Salinispora tropica*) with duplicated parts of the system. *L. ferrooxidans *contains two copies of the entire system (L1, L2), which are identical in terms of genetic context, but very different in their sequence (Pup in L2 even lacks the terminal GGE). The phylogenetic tree was calculated using PhyML [61] and displayed with iTOL [62] from MUSCLE-aligned [63] and GBLOCKS-refined [64] sequences.

Actinobacteria form a large and diverse phylum with many members living in close association with eukaryotic hosts as either pathogens (*Mycobacterium *spp.) or symbionts (nitrogen-fixing or gastrointestinal species) [15,16]. Phylogenetic analysis identified actinobacteria as one of the earliest prokaryotic lineages. They are known to share traits with eukaryotes [17]. For example, like eukaryotes they encode single-chain eukaryotic-like fatty-acid synthase (FASI; in addition to the dissociated bacterial FASII enzymes) [18], actinomycetes form exospores and mycobacteria produce sterols [17]. Another eukaryotic-like feature is the existence of proteasomes in actinobacteria in addition to the typical bacterial-like compartmentalizing protease complexes (Clp proteases [19], FtsH [20], Lon [21], but not HslUV) [22]. These bacterial proteases are architecturally related to the proteasome but of only very distant homology [23]. It is still a matter of debate how actinobacteria came by their proteasomes. One theory proposes horizontal transfer of the corresponding proteasomal genes from archaea or eukaryotes [22]. In contrast to that, others suggest that the actinobacterial proteasome represents an ancestral form, based on their hypothesis that eukaryotes and archaea derived from actinobacteria [24]. Irrespective of the suggested evolutionary scenarios, the fact remains that no bacterial proteasomes were found outside the actinobacterial phylum beyond a few sporadic cases in other lineages like, for example, nitrospirae [25]. The pupylation machinery of nitrospirae, in fact, was speculated to originate in Acidimicrobiales by horizontal gene transfer [26], which seems to be supported by the recent availability of such a genome [27] (Figure 2).

The post-translational modification Pup that recruits proteins for degradation by bacterial proteasomes is functionally related to the eukaryotic ubiquitin (Ub) tag without showing any sequence or structural homology (Figure 1). Both proteins are small (below 10 kDa), both carry a di-glycine motif either at the very carboxyl terminus (Ub) or at the penultimate position (Pup) and both are attached to the amino group of lysine side chains in target proteins via an isopeptide bond [5,6,9]. However, the enzymatic pathways for attachment are different. Ub is conjugated to substrates in a multi-step reaction involving a cascade of three enzymes [2], the Ub activating enzyme E1, the Ub conjugating enzyme E2 and one of the many Ub-protein ligase E3s that form the isopeptide-bond between a substrate lysine and Ub. Ligation of Pup to target lysines on the other hand is carried out by a single enzyme, the Pup ligase PafA (proteasome accessory factor A) [9]. In all mycobacteria and many other actinobacteria, preparation of Pup by another enzyme (Dop, deamidase of Pup) must, however, occur before the actual ligation [9]. This can be likened to the processing of the Ub-precursor to reveal the carboxy-terminal di-glycine motif.

Ub adopts a defined three-dimensional structure in solution referred to as the β-grasp fold [28]. In contrast, Pup is mostly unstructured in its free, unbound form [29-31]. It has been noted that the carboxy-terminal half of Pup exhibits a pattern of hydrophobic and hydrophilic residues typical of coiled-coil formation, and NMR analysis revealed signals from weak helix formation in that part of the protein [29]. It was therefore suggested that Pup interacts with the coiled-coil domains that extend from the surface of the proteasomal ATPase ring to form a shared coiled-coil. The crystal structure of a carboxy-terminal Pup fragment with a fragment of the Mpa (mycobacterial proteasomal ATPase) coiled-coil domain confirmed this hypothesis, demonstrating that, indeed, Pup can adopt such a structure when associated with binding partners [32]. Engagement of a pupylated substrate into the proteasome occurs by means of the unstructured amino-terminal portion of Pup that is first threaded into the Mpa central pore and then translocated into the proteasomal degradation chamber [10,33].

## Pupylation gene locus and its spread in bacterial species

In mycobacteria and most other actinobacteria, the prokaryotic ubiquitin-like protein Pup is encoded directly upstream of the 20S proteasomal subunit genes (*prcB, prcA*) (Figure 2b). The *pup *gene is usually preceded by the *dop *gene, while the Pup ligase gene, *pafA*, is located downstream of the proteasomal subunit genes, in some cases separated by multiple functionally unrelated open reading frames. The proteasomal ATPase gene (referred to as *mpa *in mycobacteria or as *arc *in other actinobacteria) is also found in close proximity, located in most cases in a separate operon upstream of *dop*.

All *pup *genes encode small proteins ranging from 60 to 70 residues in length. As mentioned, despite the functional analogy, Pup does not exhibit any sequence homology to ubiquitin except for the di-glycine motif near the carboxyl terminus. Interestingly, Pup sequences end in Gly-Gly-Gln (PupQ) or Gly-Gly-Glu (PupE) depending on the bacterial species, with some organisms even featuring two *pup *genes, one for PupQ and one for PupE. All mycobacterial Pup sequences, however, invariantly feature the GGQ terminus.

Some actinobacteria - for example, corynebacteria - possess the pupylation genes but lack the proteasomal subunit genes (Figure 2b). In those genomes, *pup *directly precedes the Pup ligase gene *pafA*. Interestingly, these genomes nevertheless maintain the proteasomal ATPase gene. Furthermore, the ATPases from organisms without proteasome subunit genes do not harbor the carboxy-terminal proteasome-interaction motif [34]. This implies that, at least in these bacteria, the proteasomal ATPase plays a different role in the pupylation system than targeting pupylated substrates for proteasomal degradation.

Indeed, subjecting the pupylation-characteristic enzymes to phylogenetic analysis (without including proteasomal subunits) reveals a clustering different from the common phylogenetic relationships of the respective bacteria (Figure 2a). For example, the pupylation machinery of proteasome-harboring Corynebacterineae forms a tight cluster closely related to the enzymes of many representatives of related clades. However, they are only weakly linked to the proteins of the genus *Corynebacterium*, although this genus belongs to the same bacterial suborder. Interestingly, the enzymes of proteasome-lacking organisms exhibit more sequence variation, only matched by Nitrospirae exponents. These features could indicate directional evolution after a dramatic genomic change, as, for example, the loss of the proteasome or the horizontal gain of the entire pupylation system.

## The enzymes of the pupylation pathway

**Figure 3:**
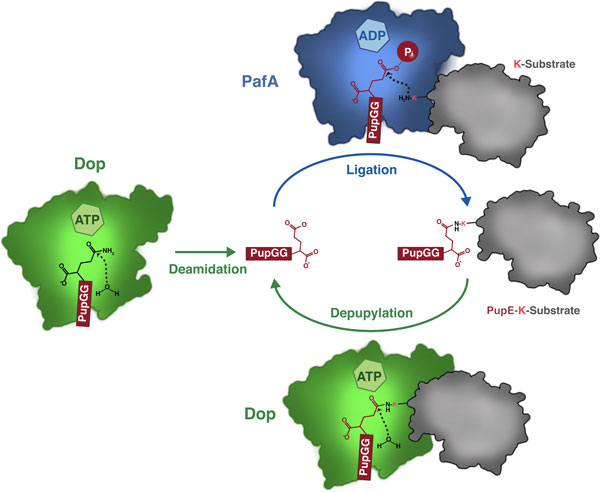
**The pupylation/depupylation cycle in mycobacteria**. Dop (green) renders PupQ ligation-competent by deamidating the carboxy-terminal glutamine to glutamate. The Pup ligase PafA (blue) then performs the isopeptide-bond formation between the γ-carboxylate of Pup's carboxy-terminal glutamate and the ε-amino group of a substrate (grey). Dop also carries out the specific cleavage of the isopeptide-bond in a pupylated substrate, depupylating the tagged substrate protein. Pup (red) has been placed in the proposed Pup-binding groove of Dop or PafA with its carboxy-terminal residue pointing into the active site.

**Figure 4:**
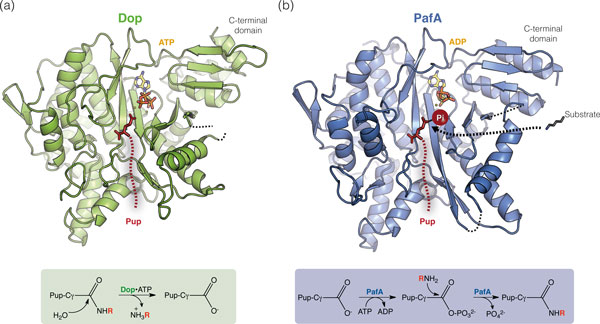
**Dop and PafA are close structural homologs but catalyze distinct reactions**. **(a,b) **The structures of Dop (a) and PafA (b) are shown in cartoon representation with ATP/ADP displayed in stick format. The catalyzed reactions are shown below the corresponding enzyme. For deamidation, R denotes a hydrogen; for depupylation or ligation, R denotes the protein lysyl moiety. Pup (dotted red line) is represented as binding in the putative Pup-binding groove with its carboxy-terminal glutamate (red, sticks) placed in the active site according to the position of glutamate in glutamyl-cysteine ligase (PDB: 2gwd). In PafA, Pup is displayed in its activated form corresponding to the phosphorylated intermediate shown in the scheme below (Pi is indicated by a red sphere).

During pupylation an isopeptide bond is formed between the small protein tag Pup and a lysine residue of the target protein [5,6,9] (Figure 1). In mycobacteria, this involves the sequential action of two homologous enzymes, the deamidase Dop (deamidase of Pup) and the Pup ligase PafA (proteasome-accessory factor A) [9] (Figure 3). First, Pup is rendered coupling-competent by deamidation of its carboxy-terminal glutamine to glutamate through the action of Dop (Figure 4a, reaction scheme). In the second step, the enzyme PafA catalyzes the formation of an isopeptide bond between Pup's carboxy-terminal glutamate and the ε-amino group of a lysine residue on the substrate protein (Figure 4b, reaction scheme). Deletion strains of *Mtb *or *M. smegmatis *lacking the *pafA *or the *dop *gene are unable to produce pupylated target proteins [5,35,36], demonstrating that only one ligase and deamidase are responsible for the pupylation pathway. NMR analysis revealed that the side chain carboxylate of Pup's carboxy-terminal glutamate forms the linkage to the substrate-lysine [37]. This is different from the linkage of ubiquitin, which occurs via the terminal carboxylate of the end-standing glycine. The entire pupylation pathway has been reconstituted *in vitro *for *Mtb *and *Corynebacterium glutamicum *[9,12,38].

A key feature of regulatory post-translational modification mechanisms is their reversibility [1]. In eukaryotes ubiquitination is reversed by the action of deubiquitinases breaking the isopeptide linkage between ubiquitin and target lysines [39-41]. It was shown that Dop, the enzyme responsible for rendering Pup ligation-competent, also acts as a depupylase, removing Pup from substrates by specific cleavage of the isopeptide bond between Pup and the substrate [11,12] (Figure 4a, reaction scheme). This explains why some actinobacteria encoding Pup with a carboxy-terminal glutamate (like *C. glutamicum*), bypassing the need for deamidation to become coupling-competent, still maintain a *dop *gene (Figure 2b). Interestingly, the proteasomal ATPase Mpa enhances depupylation *in vitro *[12], likely by making the isopeptide bond to target proteins more accessible. This might be one reason the ATPase gene is maintained in actinobacteria that possess the puplyation enzymes in absence of the proteasome core particle (Figure 2b). In summary, this suggests a role for Pup that is independent of degradation - for example, as a regulatory tag analogous to mono-ubiquitination.

## Structure and mechanism of the enzymes involved in pupylation and depupylation

The Pup ligase PafA and the deamidase/depupylase Dop are close structural homologs [38] and are related to the carboxylate-amine ligase superfamily [25] (Figure 4). Bioinformatic analysis of non-ribosomal bacterial amidoligases suggests that Pup-ligase PafA is an evolutionary derivative of glutamine synthetases [42]. In the same study, it was also proposed that the Pup tag itself might have evolved from ribosomally synthesized and later cyclized bioactive peptides produced, for example, as defensins (like marinostatin). The authors reason that, like these peptide metabolites, Pup is small and disordered. Instead of cyclization, however, ligation in the case of Pup occurs as conjugation to other proteins.

Both Dop and PafA are globular in shape and consist of two tightly interacting domains, a large amino-terminal domain of about 400 residues and a small carboxy-terminal domain of about 70 residues [38] (Figure 4). The amino-terminal domain is homologous to the carboxylate-amine ligase family [25]. It consists of a curved, six-stranded β-sheet that packs against a cluster of helices. The active site is located on the concave side of the β-sheet with ATP bound in a deep pocket at one end of the sheet. A defined, well-conserved groove leads into the active site at the opposite end and has been proposed as the Pup-binding groove [38]. In PafA, this would place the carboxy-terminal glutamate of Pup directly into the active site at the position where glutamate is found in the related glutamine synthetase enzymes. The open access to the active site on the shallow β-sheet cradle allows accommodation of a diverse range of substrate proteins of varying sizes and oligomeric states.

Despite featuring highly homologous folds, Dop and PafA catalyze separate reactions with opposing activities [9,11,12] (Figure 3). The Pup ligase PafA forms the isopeptide bond between the protein lysyl moiety and Pup's carboxy-terminal glutamate [9], while Dop removes the protein lysyl moiety (or ammonia) from the Pup carboxy-terminal side chain [9,11,12]. Formation of the isopeptide bond by PafA requires turnover of ATP to ADP [9]. It has been demonstrated that the reaction proceeds through a γ-glutamyl-phosphate mixed anhydride intermediate that is formed on the carboxy-terminal glutamate of Pup to activate it for nucleophilic attack by the lysine side chain [43]. During deamidation/depupylation, activation is not needed; thus, no ATP turnover takes place [9,12]. In both reactions, a nucleophilic attack must occur on the carbonyl-carbon of Pup's carboxy-terminal glutamine/glutamate side chain by either water, in the case of Dop, or the ε-amino group of lysine, in the case of PafA. A loop between two of the strands in the β-sheet cradle is ideally located to provide catalytic assistance and contains a conserved aspartate that has been proposed as the catalytic base that activates the nucleophile (water or lysine side chain) [38]. A mechanistic study on Dop also identified this aspartate as a crucial catalytic residue and proposes that it might even act as a direct nucleophile, forming a covalent intermediate with Pup [44].

## Role of the Pup-proteasome system in actinobacteria and for pathogenicity of *Mtb*

Actinobacteria carry the PPS in addition to a subset of the usual bacterial energy-dependent proteases (Clp proteases, the membrane-associated FtsH, Lon) [45]. The ATP-dependent protease profiles differ between the individual members [45]. Proteasome subunit-bearing bacteria generally do not have HslUV, another compartmentalizing protease complex, but they may code for Lon protease (leptospirilli present an exception and carry both). For example, *Mtb *lacks both HslUV and Lon protease, while *M. smegmatis *retains a *lon *gene. Disruption of 20S proteasome subunit genes in *M. smegmatis *as well as in *Streptomyces coelicolor *and *lividans *resulted in mutant strains with the same growth behavior in standard liquid aerobic culture as their parent strains [46-48]. Even in *Mtb*, where both HslUV and Lon are lacking, removal of the 20S subunits has only minor effects on growth under standard culture conditions [13]. Likewise, disrupting other genes of the pupylation gene locus does not result in a significant change in growth phenotypes in standard liquid culture [14,48,49]. This suggests that the PPS might provide an advantage under specific environmental conditions encountered by the bacteria or during the switch to a different state in their life cycle. One organism facing particular challenges during its life cycle is the human pathogen *Mtb*.

The cellular machinery of *Mtb *is optimized to persist in one of the most inhospitable niches in humans, the macrophage [50]. While inside the host, *Mtb *faces multiple chemical stresses, such as a drop in pH, reactive oxygen species and increased toxic ion concentration [51,52]. However, a primary killing mechanism employed by infected macrophages is the production of highly reactive nitrogen intermediates (RNIs) produced by the interferon-γ-inducible nitric oxide synthase (iNOS, NOS2) [53]. *Mtb *lacking the 20S subunits is highly susceptible to nitrosative stress *in vitro *and silencing of the *Mtb *20S proteasome after inhalation-infection of mice leads to lung bacterial counts reduced by two or three orders of magnitude [13]. A transposon mutagenesis screen aimed at finding targets that contribute to making *Mtb *resistant to nitrosative stress identified mutants in the PPS gene locus in the *mpa *and *pafA *genes [14]. This points to a role of the PPS in helping *Mtb *cope with RNIs, perhaps by clearing damaged proteins. However, the role of the PPS must go beyond mere defense against nitrosative stress, because interferon-γ-deficient mice that are unable to induce nitrosative stress still show significantly increased survival when infected with a proteasome-depleted *Mtb *strain versus wild-type *Mtb *[13]. Furthermore, proteomic studies on standard *in vitro *cultures of *Mtb *and *M. smegmatis *identified around 700 pupylation targets associated with a wide range of cellular functions, including a large number from intermediary or lipid metabolism [54-56]. This suggests that the effect of the PPS on *Mtb *survival in the host could be multicausal and might be related to more than one set of substrates. Investigation of transcriptional changes in *Mtb *with a defective PPS identified changes in the genes of two regulons, the zinc-uptake regulon and a copper-responsive regulon [57]. The changes indicated that the PPS knockouts have lower intracellular levels of zinc and copper, though it is unclear how the PPS affects these levels. Furthermore, no direct link to pathogenicity of *Mtb *could be made.

Although a lot has been learned about pupylation, its mechanism, *in vivo *effects and spectrum of substrates, the ultimate question of what role the PPS plays for *Mtb *pathogenicity remains yet unanswered.

## Outlook

The discovery of pupylation established that bacteria use macromolecular tagging in their post-translational modification repertoire. The functional analogies to ubiquitination, yet separate evolutionary origin and distinct modification pathway, have sparked great interest in this system. The fact that one of the deadliest bacterial pathogens known to mankind, *Mycobacterium tuberculosis *(*Mtb*), makes use of the pupylation pathway to help overcome the immune defense of its host, adds the compelling element of biomedical relevance to an exciting biological system.

But this is also where several questions still await answering. By what mechanism does pupylation impact the virulence of *Mtb*? And why do non-pathogenic members of this phylum maintain this system in their genome? It is doubtful whether these answers can be obtained by investigation of lab cultures grown under standard conditions, where the PPS is verifiably not required. Rather, it is now crucial to investigate this question under conditions where pupylation contributes to survival. In this context, it will be critical to obtain information about the nature of the pupylome from *Mtb *inside activated macrophages. This might shed light on which pupylation substrates or groups of substrates provide the decisive advantage. Another open question is the role of pupylation in the context of proteasomal degradation. To what degree is pupylation truly a degradation tag and to what degree does it act as a regulatory tag? Answers to these questions might come from investigating actinobacterial members that have lost the proteasomal genes and hence the degradative branch of pupylation. A somewhat puzzling observation has been the existence of just one ligase for attaching Pup to a large range of target proteins. How are substrates selected for pupylation? It remains to be seen whether additional cellular factors impose some selectivity and regulation on the system.

With the insights already gained about pupylation in mycobacteria over the past few years an excellent foundation has been laid on which to build future experimental approaches. One important route is certainly also the pursuit of the PPS as a drug target to combat, in particular, the emerging multi-drug resistant *Mtb *strains [58-60].
